# Convolutional Neural Network Model for Variety Classification and Seed Quality Assessment of Winter Rapeseed

**DOI:** 10.3390/s23052486

**Published:** 2023-02-23

**Authors:** Piotr Rybacki, Janetta Niemann, Kiril Bahcevandziev, Karol Durczak

**Affiliations:** 1 Department of Agronomy, Faculty of Agronomy, Horticulture and Bioengineering, Poznań University of Life Sciences, Dojazd 11, 60-632 Poznań, Poland; 2 Department of Genetics and Plant Breeding, Faculty of Agronomy, Horticulture and Bioengineering, Poznań University of Life Sciences, Dojazd 11, 60-632 Poznań, Poland; 3 Agricultural College of Coimbra (ESAC/IPC), Research Centre for Natural Resources, Environment and Society (CERNAS), 3045-601 Coimbra, Portugal; 4 Department of Biosystems Engineering, Faculty of Environmental and Mechanical Engineering, Poznań University of Life Sciences, Wojska Polskiego 50, 60-637 Poznań, Poland

**Keywords:** winter rapeseed, *Brassica napus* L., seed quality, Python, machine learning, CNN

## Abstract

The main objective of this study is to develop an automatic classification model for winter rapeseed varieties, to assess seed maturity and damage based on seed colour using a convolutional neural network (CNN). A CNN with a fixed architecture was built, consisting of an alternating arrangement of five classes Conv2D, MaxPooling2D and Dropout, for which a computational algorithm was developed in the Python 3.9 programming language, creating six models depending on the type of input data. Seeds of three winter rapeseed varieties were used for the research. Each imaged sample was 20.000 g. For each variety, 125 weight groups of 20 samples were prepared, with the weight of damaged or immature seeds increasing by 0.161 g. Each of the 20 samples in each weight group was marked by a different seed distribution. The accuracy of the models’ validation ranged from 80.20 to 85.60%, with an average of 82.50%. Higher accuracy was obtained when classifying mature seed varieties (average of 84.24%) than when classifying the degree of maturity (average of 80.76%). It can be stated that classifying such fine seeds as rapeseed seeds is a complex process, creating major problems and constraints, as there is a distinct distribution of seeds belonging to the same weight groups, which causes the CNN model to treat them as different.

## 1. Introduction

Rapeseed (*Brassica napus* L.) is the second largest source of vegetable oil in the world, after soya, and the first on the European continent [1,2,3,4,5,6]. According to a report by the United States Department of Agriculture Foreign Agricultural Service [7] global rapeseed seeds production for the 2021/2022 season amounted to 73.86 million tones. Eurostat [8] and the International Grains Council report, that European Union countries produced over twenty million tons of seeds. This means that EU rapeseed production, although 9% lower than the record in 2020/2021, is more than 5% higher compared to the average of the last five years [8].

The value of rapeseed seeds, which are a raw material in the oil industry, is strictly dependent on both the harvesting technology (maturity, amount of damage) and the conditions and method of postharvest handling, especially drying, cleaning, transport, and storage. Therefore, from the technological value point of view, an immensely important problem in rapeseed production is the reduction of seed quality losses. Mechanical damages also cause the initiation of unfavorable chemical and biological transformations in the seeds, which result, inter alia, from their morpho-anatomical structure and chemical composition [2,3,9,10,11].

Mechanical damage to rapeseed seeds most often occurs during harvesting and transport, where there is an interaction of mechanical forces derived from the speed at which moving machinery parts hit the seeds, according to Wang et al. [12]. This speed, along with moisture, as well as seed size, maturity, drying time, and storage conditions, has a decisive influence on the number and degree of damage. The inappropriate exploitation of the machinery used largely contributes to this type of damage. As research shows, up to 15% of micro- and macro-damage to seeds occurs during harvesting because of an inadequate adjustment of individual harvester components, considering the maturity and moisture of the harvested crop.

Rapeseed seeds’ resistance to mechanical damage also depends significantly on their moisture. Higher water content means greater seed flexibility and greater susceptibility to deformation. On the other hand, at low water content, seeds become hard and brittle, and external stresses contribute to the formation of cracks and halving. According to Stępniewski et al. [13] and Szwed et al. [14] initially, as the amount of water rises, the dynamic strength of seeds increases, and then decreases. Seeds with a moisture of less than 7% are the least resistant, and their harvesting and transport (especially pneumatic transport) cause major damage.

The outer surface of the seed has numerous pores and holes that increase the surface area of the seed coat. They also increase the coefficient of wall friction, which affects the contact between seeds and the working elements of the threshing unit. In the polar part of the seed, there is a dent caused by the radicle located under the seed coat. The fact that the seed is not a homogenous, smooth sphere, with a sphericity index range from 0.85 to 0.9 suggests, that there are areas on the seed coat surface that are especially susceptible to cracking [15,16,17,18]. The edge of the seed coat, cracked under contact stresses, is nonhomogeneous and jagged, influenced by the unevenness of the seed coat.

The deep neural networks (DNN) and the convolutional neural networks (CNN) method is a modern and dynamically developing tool used to solve different tasks of multilevel complexity, e.g.,: image analysis and objects recognition [19,20,21], face and action recognition [22], drivers monitoring [23,24,25], quality assessment of products, agricultural produce, and other biological materials [26,27]. DNN significantly improved speech recognition accuracy [28,29,30], and related tasks, such as machine translation [31], natural language processing [32], or generating sound [33]. Models generated by DNN play an important role in understanding the genetics of such illnesses as autism and spinal muscular atrophy [34]. They are also used in medical imaging for detecting cancer of the skin [35], the brain [36], and the breasts [37]. Deep neural networks are also used in robotics for programming manipulators [38], ground robots motion path planning [39], visual navigation [40], aircraft control [41], and autonomous vehicle trajectory control [42].

The possibility of using CNN and image analysis to assess the quality of food products, roots, and the identification of weeds, diseases, and pests of crops are currently the aim of interest for many researchers [43,44,45,46,47]. Computer image analysis becomes one of the main techniques used in agriculture to assess seeds and grains in terms of quality losses, quantifying their degree of mechanical damage, maturity stage, disease infestation, or contamination with other plant species [48]. Due to its noninvasive character and increasing computing power, machine image analysis has significant advantages over labour-intensive and costly methods that destroy the material being assessed [49,50]. Computer techniques enable the implementation of precision agriculture technology to agronomic treatments, balanced fertilization, and strip spot application of plant protection products [51,52].

The use of computer image analysis to assess the quality of seeds, leaves, inflorescences, or even whole rapeseed plants, is presented in numerous studies. Scientists Xia et al. [53] used hyperspectral image analysis (HSI) to detect rapeseed plants’ stress to prolonged flooding of crops in water. The authors studied images of leaves of two rapeseed varieties collected during three periods of plant growth under waterlogged conditions. Kong et al. [54] used the hyperspectral imaging method with a spectral range of 384–1034 nm to detect Sclerotinia sclerotiorum on rapeseed stems, and Zhao et al. [55] on petals. The hyperspectral imaging method was also used by Zhang et al. [56] to determine the soluble protein content, the sugar content Zhang et al. [57], and Bao et al. [58], in turn, to detect glutamic acid in rapeseed leaves. Olivos-Trujillo et al. [59] used a near-infrared spectroscopy method (NIR) and image analysis to determine fat content and other qualitative parameters of rapeseed seeds. In this study, the authors presented three predictive models, of which the ANN-based (Artificial Neural Networks) model had the highest accuracy. Zhang et al. [60] used hyperspectral imaging and leaves images, in turn, to estimate quickly rapeseed seeds yield. Image analysis is also a good method to assess plants’ nutrition level, estimate the number of micro- and macro-nutrients, and a great tool to support the decision-making process of mineral fertilization in precision agriculture conditions [61]. The development of artificial intelligence and the use of CNNs in agricultural practice allows rapid and highly accurate identification of objects and non-destructive diagnostics of real-world models, including plant materials. CNN models are essential in the application of ‘Agriculture 4.0’ technology and digital data analysis. With this in mind, the authors have set themselves the main objective of developing an automatic classification model for winter rapeseed varieties, using a CNN, based on a seed maturity evaluation and seed damage expressed threw a seed coat colour. In this study, an attempt was made to develop a CNN structure, an algorithm describing this structure in order to facilitate the identification of oilseed rape seeds and their degree of damage. In agricultural practice, the ability to quickly assess the degree of seed damage is important in terms of storage and suitability for the processing industry.

## 2. Material and Research Method

### 2.1. Data Set Preparation

Seeds of three winter rapeseed varieties were used for this study, i.e., Atora F1, Californium, and Graf F1, which were obtained from Dłoń (51°41′23″ N, 17°04′10″ E) the experimental station of the Poznań University of Life Science. The experimental plots were characterized by soil quality class III, heavy soil type, and good rye complex of agricultural suitability. Mean annual temperature 9.93 °C, sum of precipitation in the whole year 553.67 mm. Seeds were cleared on the sieves and at this time all foreign bodies such as dust, soil residues, stones, and siliques were removed from the samples. Then, the seeds were stored in paper bags at room temperature (20–25 °C). Each imaged sample had a weight of 20.000 g, which allowed it to cover tightly the bottom of the plate, and for each variety 125 weight groups were prepared, with the weight of damaged or immature seeds increasing by 0.161 g in each group. The partitioning thresholds were determined by the laboratory scale range and its minimal weighed amount, which was 160 mg. There were, in turn, 20 samples of the same weight in each group (i.e., 20.000 g), but with differently spread rapeseed seeds. Imaged samples were labelled with a code containing the variety symbol and a sequence number, i.e., atora.0–atora.2499, californium.0–californium.2499, and graf.0–graf.2499. A detailed list of sample codes and seed weights are shown in Table 1.

**Figure 1:**
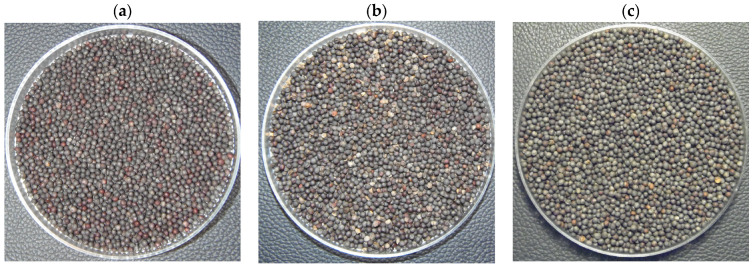
Seed samples of oilseed rapeseed varieties: (**a**) Atora F1, (**b**) Californium, (**c**) Graf F1.

Rapeseed seed images were taken with a digital camera, which had a 4288 × 3216 (14 million) pixel sensor and a 1/2.3 inch class sensor. The camera was equipped with a 36× optical zoom lens and its shortest focal length was 24 mm, corresponding to the largest aperture of 1:2.9. Seed imaging was performed at maximum zoom and the imaging surface was at a distance of 40 cm from the lens. Imaging was performed in a chamber illuminated by three light sources, at 800 lumens, with a black and non-reflective surface. The image files were stored in the camera’s internal memory, and saved in 96 dpi resolution (2139 × 1888) size in the computer’s memory (Figure 1).

### 2.2. Defining Seeds Classification Criteria

In optical object recognition and classification, it is very important to select appropriate features of the analyzed image, which should describe them unambiguously. The analyzed images of rapeseed seeds contain small-size, low-contrast objects, which was a determinant in the selection of their resolution. In the classification and recognition of seeds images of the Atora F1, Californium, and Graf F1 varieties, the basic criterion was the colour of the mature seeds or the colour of the seeds at the same weight as the immature seeds, i.e., the different weight groups were compared in pairs. When assessing the degree of seed maturity in individual rapeseed varieties, it was assumed that mature seeds suitable for long-term storage had no more than 1% of immature or damaged seeds. Therefore, in the samples analyzed, the first and second weight groups are considered mature, i.e., samples atora.0–atora.39, californium.0–californium.39, and graf.0–graf.39. These weight groups were treated as one set of the given variety, which was compared with the others considered non-compliant.

### 2.3. Experimental Set Up

In this study, the algorithms were developed in the Python 3.9 programming language using scientific computing libraries (environments) TensorFlow 2.0, Keras, Scipy, and Numpy. The TensorFlow 2.0 library is a scalable and cross-platform programming interface for running machine learning algorithms. Keras is a specialized API (Application Programming Interface) interface intended for creating neural networks, originally designed as a support class for the TensorFlow 2.0 library. SciPy, on the other hand, is an open-source Python library that is used to solve scientific and mathematical problems. It is built on the NumPy extension and allows the user to manipulate and visualize data using a wide range of high-level commands.

### 2.4. Loading and Pre-Processing a Data Set

Conceptually, an image in its simplest single-channel form (e.g., binary, monochrome, greyscale, or black and white) is a two-dimensional function f(x, y), mapping a coordinate pair to a real number that is related to the intensity (colour) of a given point. An image can have multiple channels, such as an RGB, where the colour is represented by using three channels red, green, and blue. For an RGB colour image, each pixel in the (x, y) coordinates can be represented by three tuples (Ir_x_,_y_, Ig_x_,_y_, Ib_x_,_y_). To be processed, the image f(x, y) must be digitized in spatial and amplitude terms. Spatial coordinates (x, y) digitization is called image sampling, and amplitude digitization is called grey-level quantization. The pixel value corresponding to the channel is usually represented as an integer value in the range 0–255 or a floating-point value in the range 0–1. The image is stored as a file, and there can be many different types of files. Each file usually has some data, which can be extracted as 2D multidimensional arrays for binary or greyscale images and 3D arrays for RGB colour images.

When working with rapeseed seeds images, they are loaded into NumPy arrays using the “uint8” data type (i.e., unsigned, 8-bit fixed-point numbers), which take values in the range [0, 255], which is quite sufficient for storing pixel information in RGB images. Two TensorFlow 2.0. modules will be used to prepare the data set. The first is tf.io used for loading and storing data and the second is tf.image for decoding raw content and resizing images.

**Figure 2:**
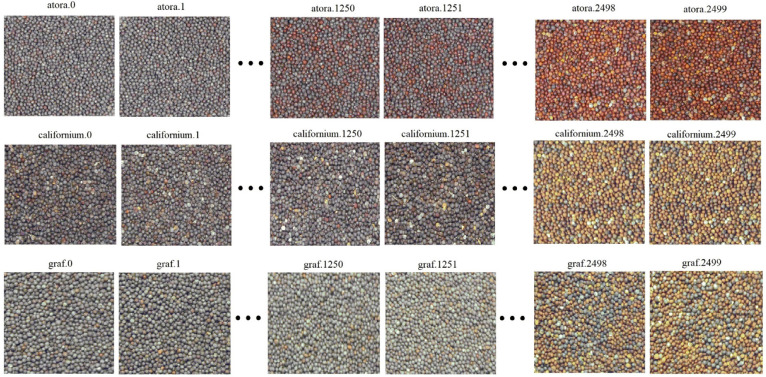
Visualization of images of rape seed samples.

Firstly, the contents of the files were checked and a list of image names of the rapeseed seeds samples was generated using the pathlib library. Then, they were visualized and sized according to code 1 added in https://github.com/piotrrybacki/seed-quality-CNN; (accessed on 19 December 2022) (Figure 2).

The list of files displayed shows, that the set of data contains 7500 images of winter rapeseed seeds, 2500 for each variety, and occupies approximately 9.5 GB. The images of the imaged rapeseed seeds were arranged in two ways depending on the type of analysis being conducted. For the recognition of rapeseed seeds variety, the images were divided into three subsets, i.e., a learning set containing 4500 samples (1500 samples from each variety), and validation and test sets containing 1500 samples (500 samples of each variety). For the seeds’ maturity assessment, each variety was in turn divided into learning sets containing 1500 samples, and validation and test sets containing 500 samples each. Depending on the type of conducted analysis, models based on the proposed CNN architecture were labelled according to the data in Table 2.

Listing 2 shows code 2 (https://github.com/piotrrybacki/seed-quality-CNN; (accessed on 19 December 2022)) for automatically copying images from the source directory to the learning, validation, and testing directories.

### 2.5. Multilayer Architecture of CNN Network

**Figure 3:**
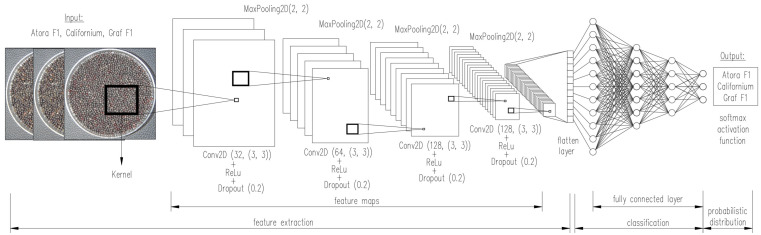
Diagram of the implemented CNN network.

The network was implemented using the Keras interface. Due to the extensive analysis of the imaged seed, the overall structure of the CNN is an alternating arrangement of five classes Conv2D (with activation function ReLu), MaxPooling2D, and Dropout. By default, the Conv2D class assumes, that the input data are compatible with the NWHC format, where N stands for the number of images in the batch group, W and H designate the width and height of the image, respectively, and C is the number of channels. As shown in Figure 3, each convolutional layer was followed by a pooling layer for subsampling, reducing the size of the feature map. MaxPoo12D class creates maximizing pooling layers. The argument pool size = 2 specifies the size of the window used to calculate the maximum value, and the strides = 1 parameter was used to configure the pooling layer. The use of the Dropout class in the analysis will allow the construction of a dropout layer for regularization, where the argument rate determines the probability of input units being dropped during network learning. When calling this layer, it is possible to regulate its operation by using a training argument, which determines if the call is to occur during learning or inference.

The input sensor was arbitrarily transformed to 200 × 200 object maps to finally produce 7 × 7 object maps just before the flattening layer. The depth of object maps gradually increases in the network from 32 to 128, while the size of object maps decreases (from 200 × 200 to 7 × 7). As the model under development uses a binary classification, the network ends with Dense layers. One with a dimension of 512 and a ReLu activation function, and the second with a dimension of 1 and a Sigmoid activation function. Listing 3 attached in https://github.com/piotrrybacki/seed-quality-CNN; (accessed on 19 December 2022) shows the programming code for the model in Figure 3.

The example algorithm automatically, considering the name of the file (sample image) sorted and copied them to the appropriate directory, from which they were then downloaded by the CNN model, depending on the type of comparison executed.

The next stage of the model under development is to plot the loss curves and the analysis and prediction accuracy values according to code 4 attached in: https://github.com/piotrrybacki/seed-quality-CNN; (accessed on 19 December 2022). 

The final stage of the analysis is to display the results of the predictions in the form of probabilities of belonging to each class (of variety or maturity) and transform them into the predicted classes using the function tf.argmax, which will search for the image with the highest probability of belonging and assign a corresponding label that is the name of the variety or maturity. This was done for the group of 10 examples in each model and both input data and predicted labels were visualized according to code 5 attached in https://github.com/piotrrybacki/seed-quality-CNN; (accessed on 19 December 2022).

## 3. Results of the Analysis

The result of the conducted analyses is a proposal for a CNN architecture and a code in Python 3.9 that enables the automatic comparison and recognition of fully mature rapeseed seed varieties and the assessment of their immaturity or damage degree. Table 3 summarizes the changes in map size depending on the layer number of the developed CNN model. As can be seen from the data, each hidden layer of the CNN network model causes maps to decrease, yielding 6,795,457 parameters in the output.

**Figure 4:**
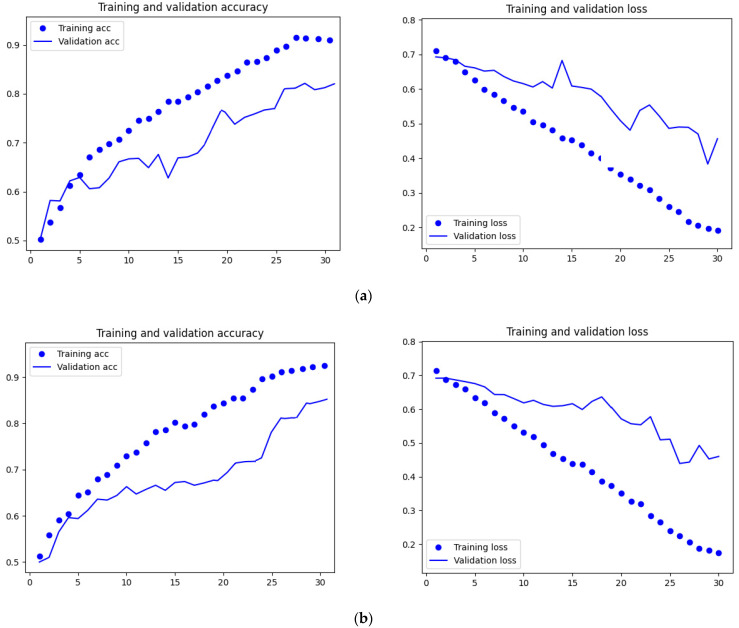

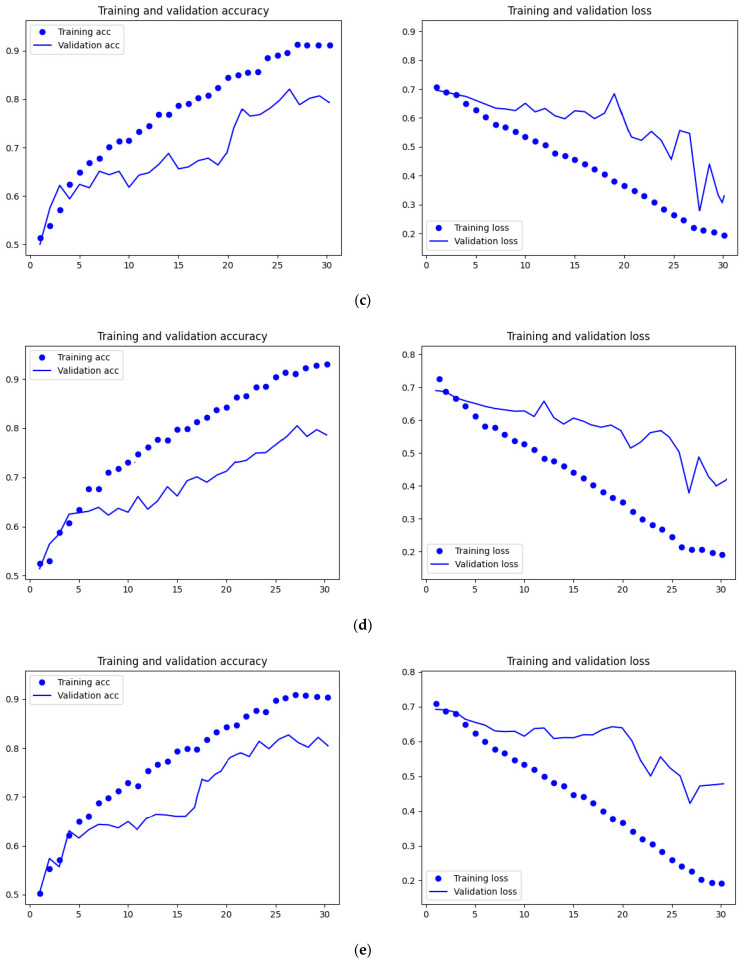

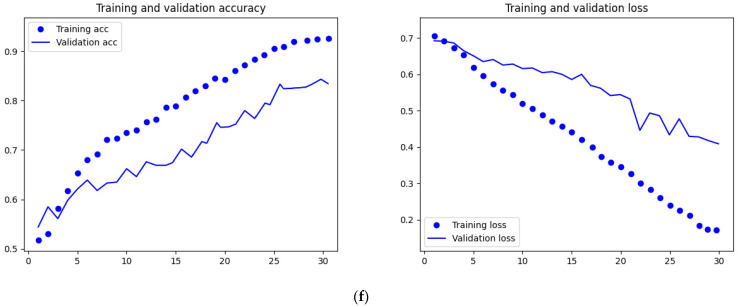
Visualization of loss function curves and learning accuracy and validation for the created CNN in models: (**a**) RAPESEEDS_AC, (**b**) RAPESEEDS_CG, (**c**) RAPESEEDS_GA, (**d**) RAPESEEDS_AQ, (**e**) RAPESEEDS_CQ, (**f**) RAPESEEDS_GQ.

The code developed for the proposed CNN architecture allowed images to be automatically sorted into training, validation, and test directories. Then, based on a random sequence, the algorithm performed unsupervised training of the individual models and their validation, the results of which are shown in Figure 4.

**Figure 5:**
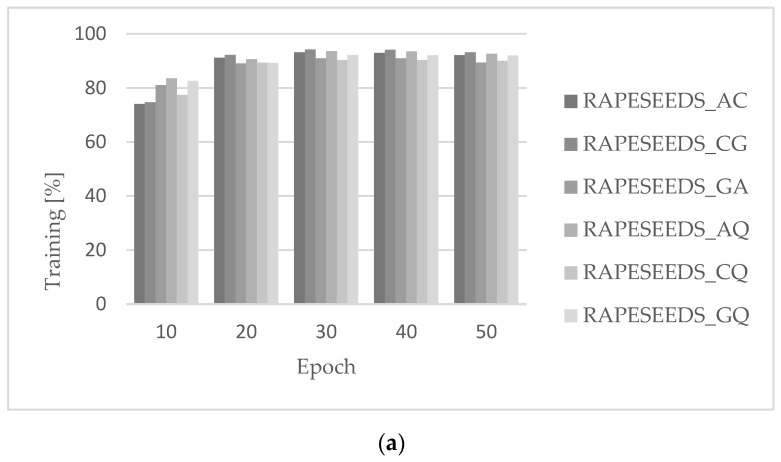

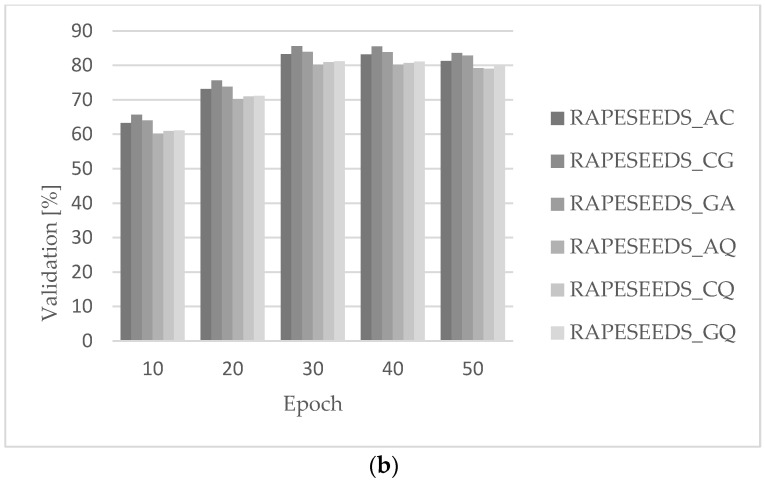
Accuracy of models depending on the number of epochs: (**a**) in the training process, (**b**) in the validation process.

The main objective of the analyses was to develop as accurate a model as possible to classify oilseed rape seeds. Therefore, the primary measure was the accuracy of the validation. As can be seen in Figure 5, this accuracy initially increased up to 30 epochs, then stabilised to 40 epochs, after which it decreased. This may be due to overtraining of the model. Therefore, 30 epochs were used as the optimal value, which is the solution to the problem.

As presented in Table 4, the accuracy of model validation ranged from 80.20% to 85.60%, with an average of 82.50%. Higher accuracy was obtained when classifying mature seed varieties (average of 84.24%) than when classifying the degree of maturity (average of 80.76%). The highest accuracy (85.60%) was obtained for the RAPESEEDS_CG model classifying mature seeds of the Californium and Graf F1 varieties, and the lowest accuracy (83.24%) for the RAPESEEDS_AC classifying mature seeds of the Atora F1 and Californium varieties. On the other hand, when assessing seeds maturity the highest accuracy (81.17%) was obtained for the RAPESEEDS_GQ model classifying Graf F1 variety, and the lowest (80.20%) for the RAPESEEDS_AQ model classifying the maturity of the Atora F1 variety.

**Figure 6:**
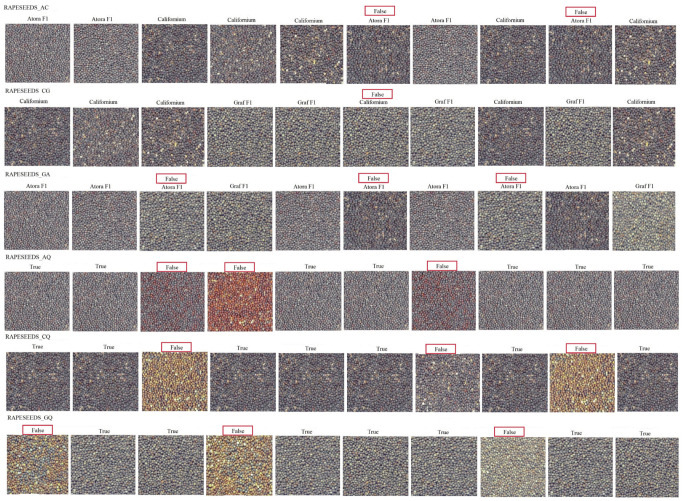
Input of oilseed rape seed images with their predicted labels. Red box indicates incorrect model reading.

The final result of the conducted analysis was to display, according to code 5, the result of the predictions in the form of probabilities of belonging to each class. The developed algorithm searched for the image with the highest probability of belonging and assigned the corresponding label, which is the name of the variety in the models: RAPESEEDS_AC, RAPESEEDS_CG, RAPESEEDS_GA, and for maturity in models RAPESEEDS_AQ, RAPESEEDS_CQ, RAPESEEDS_GQ a conventional label was “True” meaning mature seeds, or “False” for immature seeds. This was done for the group of 10 examples, in each model, and both input data and predicted labels were visualized (Figure 6). For four models (RAPESEEDS_GA, RAPESEEDS_AQ, RAPESEEDS_CQ, RAPESEEDS_GQ) out of 10 samples three were misidentified, one model (RAPESEEDS_CG) misidentified one imaged sample and one model (RAPESEEDS_AC) misidentified two samples.

## 4. Discussion

The identification and classification of rapeseed and cereal seeds have become an important part of their storage and further processing, where information on their type and quality is required. Seed classification of rapeseed varieties Bristol, Californium, Dexter, Finesse, Licord, Orkan, and Valeska was conducted by Kurtulmus and Ünal [62] using algorithms programmed in Python 2.7 language and the Scipy, Numpy, and Scikit-image environments. Using various prediction methods, they achieved an overall classification accuracy rate of 99.24%, claiming that it was even possible to achieve 100.00% model accuracy. However, such an accuracy rate is not recommended in machine learning and computer image analysis, due to the danger of overtraining the model. Research on the classification of rapeseed seeds has also been conducted by Zou et al. [63], using the potential of the visible, near-infrared spectra and Back Propagation in Neural Network BPNN, proposing a model with 100.00% accuracy. Sun et al. [64] in turn used CNN to recognize rapeseed plants in the field. They applied the method of increasing hidden convolutional layers and its impact on model accuracy. The authors showed, that increasing hidden layers does not significantly improve the accuracy of the model, obtaining the highest average recognition accuracy of 93.54%, and the minimum value of the loss function of 0.206 with three convolutional layers. Jung et al. [65], on the other hand, applied three CNN architectures to recognize rapeseed in early growth stages with 10°, rotating plant images, achieving validation accuracy ranging from 13.04 to 88.89%, with an average of 58.34%. Comparing those results to the model proposed in this study, which has an average validation accuracy of 82.50%, it can be concluded, that it meets expectations in terms of the accuracy of rapeseed seeds recognition. According to the research of Ni et al. [66] on the classification of corn seeds and Lin et al. [67] on the classification of rice seeds, it is possible to obtain higher accuracy (over 90%) with larger research objects, as it is easier to analyze their texture.

Zhang et al. [68] proposed a CNN-based algorithm for citrus fruit detection, quality classification and automatic identification of the five most common diseases. The authors tested several state of the network architectures for their performance on a set of 1524 images taken under field conditions from different orchards at different time intervals, scales, angles, and lighting conditions. They obtained fruit identification precision and accuracy of 87.2% and 89.0%. Bernardes et al. [69] used CNN methods to discriminate between Fusarium head blight (FHB)-infected seeds of wheat cultivar TBIO Toruk. The models achieved 99% accuracy in detecting FHB in seeds. These results suggest the potential of imaging technology and deep learning models for accurate seed classification.

Howard et al. [70], on the other hand, used a CNN-based model architecture, called MobileNets, for object detection, geolocalisation, fine structure classification and face recognition, while Hamid et al. [71] in their study used the MobileNetV2 spline neural network, to classify 14 different seed classes, and its accuracy was 98% and 95% in the training and test sets, respectively. The MobileNetV2 model of Albarrak et al. [72] also used a dataset containing eight different classes of date fruit in their study, achieving 99% accuracy. The proposed model was also compared with other existing models such as AlexNet, VGG16, InceptionV3, ResNet, and MobileNets.

## 5. Conclusions

This study proposes an automatic classification model for winter rapeseed seeds of three varieties and the assessment of their maturity degree based on colour contrast using CNN. A CNN with a fixed architecture was built, consisting of an alternating arrangement of five classes Conv2D, MaxPooling2D, and Dropout, for which in the Python 3.9 programming language. Using scientific computational environments TensorFlow 2.0, Keras, Scipy, and Numpy, a computational algorithm was developed, creating six models depending on the type of input data. The algorithm proposed in this study described with a code, allows the number of classes to be changed smoothly and the number of images copied to the training, validation and test directories to be changed and randomly, making data analysis much easier.

The validation accuracy of models presented in this study ranged from 80.20% to 85.60%, with an average of 82.50%. Higher accuracy was obtained when classifying mature seed varieties (average of 84.24%) than when classifying the degree of their maturity (average of 80.76%) within a single variety. This is due to the fact, that immature or damaged seeds of the varieties tested did not differ significantly in colour. After the damage to the seed coat, the seeds were a similar yellow colour. These results can be seen in Figure 6, where for four models, out of 10 samples three were misidentified, one model misidentified one imaged sample and one misidentified two samples. It should be added that when it comes to varieties classification, all samples were from the same weight groups.

As a conclusion, it can be stated that classifying such fine seeds as rapeseed seeds is a complex process, creating major problems and constraints, as there is a distinct distribution of seeds belonging to the same weight groups, which causes the CNN model to treat them as different. With this in mind, it is advisable to continue research and analysis on a vision-based seed classification model. The proposed model will be extended to classify seeds based on their texture. Analysis based on two criteria will significantly increase the accuracy of the model.

## Figures and Tables

**Table 1:** Weight group of the imaged three winter rape seed samples (rape and unripe) with the respective weights.

Seed Weight [g]				Research Sample	
Weight Group	Ripe Seeds	Unripe Seeds	Atora	Californium	Graf
1	20.000	0.000	atora.0–atora.19	californium.0–californium.19	graf.0–graf.19
2	19.839	0.161	atora.20–atora.39	californium.20–californium.39	graf.20–graf.39
3	19.677	0.323	atora.40–atora.59	californium.40–californium.59	graf.40–graf.59
4	19.516	0.484	atora.60–atora.79	californium.60–californium.79	graf.60–graf.79
5	19.355	0.645	atora.80–atora.99	californium.80–californium.99	graf.80–graf.99
6	19.194	0.807	atora.100–atora.119	californium.100–californium.119	graf.100–graf.119
7	19.032	0.968	atora.120–atora.139	californium.120–californium.139	graf.120–graf.139
8	18.871	1.129	atora.140–atora.159	californium.140–californium.159	graf.140–graf.159
9	18.710	1.290	atora.160–atora.179	californium.160–californium.179	graf.160–graf.179
10	18.548	1.452	atora.180–atora.199	californium.180–californium.199	graf.180–graf.199
11	18.387	1.613	atora.200–atora.219	californium.200–californium.219	graf.200–graf.219
…	…	…	…	…	…
115	1.612	18.388	atora.2280–atora.2299	californium.2280–californium.2299	graf.2280–graf.2299
116	1.450	18.550	atora.2300–atora.2319	californium.2300–californium.2319	graf.2300–graf.2319
117	1.289	18.711	atora.2320–atora.2339	californium.2320–californium.2339	graf.2320–graf.2339
118	1.128	18.872	atora.2340–atora.2359	californium.2340–californium.2359	graf.2340–graf.2359
119	0.967	19.033	atora.2360–atora.2379	californium.2360–californium.2379	graf.2360–graf.2379
120	0.805	19.195	atora.2380–atora.2399	californium.2380–californium.2399	graf.2380–graf.2399
121	0.644	19.356	atora.2400–atora.2419	californium.2400–californium.2419	graf.2400–graf.2419
122	0.483	19.517	atora.2420–atora.2439	californium.2420–californium.2439	graf.2420–graf.2459
123	0.321	19.679	atora.2440–atora.2459	californium.2440–californium.2459	graf.2440–graf.2459
124	0.160	19.840	atora.2460–atora.2479	californium.2460–californium.2479	graf.2460–graf.2479
125	0.000	20.000	atora.2480–atora.2499	californium.2480–californium.2499	graf.2480–graf.2499

**Table 2:** Description of arboricultural seed classification models.

Model CNN	Description
RAPESEEDS_AC	Classification of rapeseed varieties Atora F1—Californium
RAPESEEDS_CG	Classification of rapeseed varieties Californium—Graf F1
RAPESEEDS_GA	Classification of rapeseed varieties Graf F1—Atora F1
RAPESEEDS_AQ	Evaluation of rape variety Atora F1
RAPESEEDS_CQ	Evaluation of rape variety Californium
RAPESEEDS_GQ	Evaluation of rape variety Graf F1

**Table 3:** Variation of map size according to layer number of the developed CNN model.

Layer (Type)	Output Shape	Param
conv2d (Conv2D)	(None, 198, 198, 32)	896
max_pooling2d (MaxPooling2D)	(None, 99, 99, 32)	0
dropout (Dropout)	(None, 99, 99, 32)	0
conv2d_1 (Conv2D)	(None, 97, 97, 64)	18,496
max_pooling2d_1 (MaxPooling2D)	(None, 48, 48, 64)	0
dropout_1 (Dropout)	(None, 48, 48, 64)	0
conv2d_2 (Conv2D)	(None, 46, 46, 128)	73,856
max_pooling2d_2 (MaxPooling2D)	(None, 23, 23, 128)	0
dropout_2 (Dropout)	(None, 23, 23, 128)	0
conv2d_3 (Conv2D)	(None, 21, 21, 128)	147,584
max_pooling2d_3 (MaxPooling2D)	(None, 10, 10, 128)	0
dropout_3 (Dropout)	(None, 10, 10, 128)	0
flatten (Flatten)	(None, 12800)	0
dense (Dense)	(None, 512)	6,554,112
dense_1 (Dense)	(None, 1)	513

Total params: 6,795,457; Trainable params: 6,795,457; Non-trainable params: 0.

**Table 4:** Average accuracy and loss values of the training and validation process for CNN models in 30 epoch.

Model CNN	Accuracy [%]		Loss	
	Training	Validation	Training	Validation
RAPESEEDS_AC	93.11	83.24	0.18	0.38
RAPESEEDS_CG	94.19	85.60	0.17	0.43
RAPESEEDS_GA	90.90	83.88	0.19	0.32
RAPESEEDS_AQ	93.53	80.20	0.19	0.37
RAPESEEDS_CQ	90.35	80.90	0.18	0.43
RAPESEEDS_GQ	92.23	81.17	0.17	0.41
Average	92.39	82.50	0.18	0.39

## Data Availability

Data is available in https://github.com/piotrrybacki/seed-quality-CNN; (accessed on 19 December 2022).
